# (*Z*)-Ethyl 2-hydroxy­imino-2-(4-nitro­benz­yl)ethanoate

**DOI:** 10.1107/S1600536809052386

**Published:** 2009-12-16

**Authors:** Ignez Caracelli, Antonio C. Trindade, Paulo J. S. Moran, Luciana Hinoue, Julio Zukerman-Schpector, Edward R. T. Tiekink

**Affiliations:** a BioMat-Physics Department, UNESP – Univ Estadual Paulista, 17033-360 Bauru, SP, Brazil; b Instituto de Química e Biotecnologia, Universidade Federal de Alagoas, 57072-970 Maceió, AL, Brazil; c Instituto de Química, Universidade Estadual de Campinas, CP 6154, 13083-970 Campinas, SP, Brazil; d Department of Chemistry, Universidade Federal de São Carlos, 13565-905 São Carlos, SP, Brazil; e Department of Chemistry, University of Malaya, 50603 Kuala Lumpur, Malaysia

## Abstract

The title mol­ecule, C_11_H_10_N_2_O_6_, has a *Z* conformation about the C=N bond of the oxime unit. There are significant twists from planarity throughout the mol­ecule, the most significant being between the hydroxy­imino and ester groups which are effectively orthogonal with an N—C—C—O_carbon­yl_ torsion angle of 91.4 (2)°. The crystal packing features oxime–benzoyl O—H⋯O contacts that lead to chains along [010] and C—H⋯O interactions also occur.

## Related literature

For background to the synthesis of chiral hydroxy­amino­acids and hydroxy­amino­alcohols, see: Corrêa & Moran (1999 ▶); Kreutz *et al.* (1997 ▶, 2000 ▶). For related structures, see: Ramos Silva *et al.* (2004 ▶); Forsyth *et al.* (2006 ▶). For the synthesis, see: Adkins & Reeve (1938 ▶).
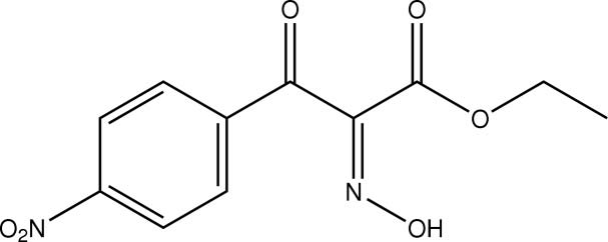


## Experimental

### 

#### Crystal data

C_11_H_10_N_2_O_6_
*M* *_r_* = 266.21
Monoclinic, 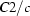

*a* = 23.2347 (7) Å
*b* = 12.0698 (6) Å
*c* = 8.9698 (4) Å
β = 106.100 (2)°
*V* = 2416.82 (18) Å^3^
*Z* = 8
Mo *K*α radiation
μ = 0.12 mm^−1^
*T* = 290 K
0.18 × 0.15 × 0.12 mm

#### Data collection

Nonius KappaCCD diffractometer
8147 measured reflections
2752 independent reflections
2004 reflections with *I* > 2σ(*I*)
*R*_int_ = 0.033

#### Refinement

*R*[*F* ^2^ > 2σ(*F* ^2^)] = 0.048
*wR*(*F* ^2^) = 0.128
*S* = 1.05
2752 reflections
189 parameters
H-atom parameters constrained
Δρ_max_ = 0.20 e Å^−3^
Δρ_min_ = −0.19 e Å^−3^

### 

Data collection: *COLLECT* (Nonius, 1999 ▶); cell refinement: *SCALEPACK* (Otwinowski & Minor, 1997 ▶); data reduction: *DENZO* (Otwinowski & Minor, 1997 ▶) and *SCALEPACK*; program(s) used to solve structure: *SIR97* (Altomare *et al.*, 1999 ▶); program(s) used to refine structure: *SHELXL97* (Sheldrick, 2008 ▶); molecular graphics: *DIAMOND* (Brandenburg, 2006 ▶); software used to prepare material for publication: *WinGX* (Farrugia, 1999 ▶), *PARST* (Nardelli, 1995 ▶) and *publCIF* (Westrip, 2009 ▶).

## Supplementary Material

Crystal structure: contains datablocks global, I. DOI: 10.1107/S1600536809052386/hg2614sup1.cif

Structure factors: contains datablocks I. DOI: 10.1107/S1600536809052386/hg2614Isup2.hkl

Additional supplementary materials:  crystallographic information; 3D view; checkCIF report

## Figures and Tables

**Table 1:** Hydrogen-bond geometry (Å, °)

*D*—H⋯*A*	*D*—H	H⋯*A*	*D*⋯*A*	*D*—H⋯*A*
O4—H4o⋯O3^i^	0.82	1.91	2.7165 (16)	166
C5—H5⋯O5^i^	0.93	2.58	3.371 (2)	144
C2—H2⋯O1^ii^	0.93	2.55	3.393 (3)	151
C11—H11a⋯O1^iii^	0.96	2.52	3.426 (5)	158

Symmetry codes: (i) 
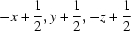
; (ii) 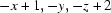; (iii) 
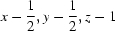
.

**Fig. 1.**
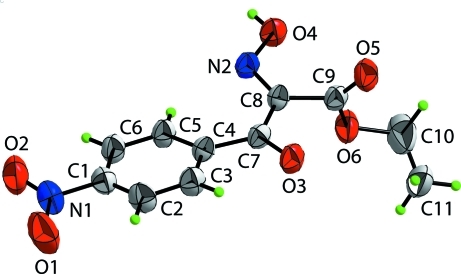
Molecular structure of (I) showing atom labelling scheme and displacement ellipsoids at the 50% probability level (arbitrary spheres for the H atoms). Only one component of the disordered ethyl group is shown for reasons of clarity.

**Fig. 2.**
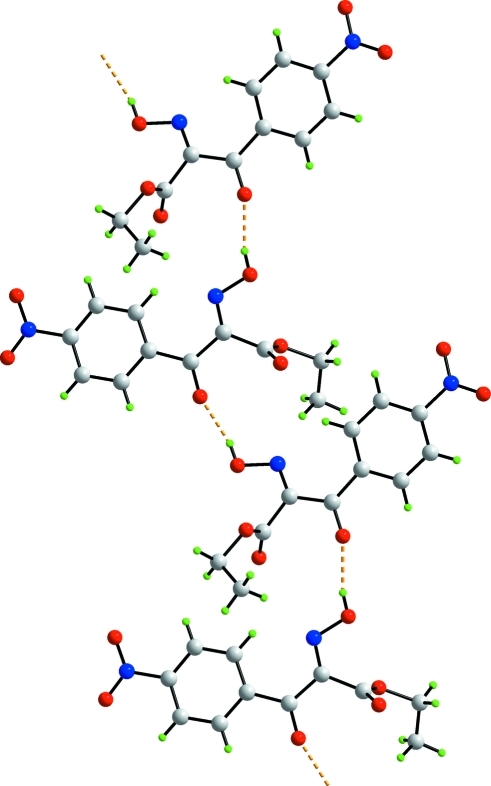
Supramolecular chain aligned along [0 1 0] in (I) mediated by oxime-O—H···O-benzoyl hydrogen bonding (orange dashed lines). Colour code: O, red; N, blue; C, grey; and H, green.

**Fig. 3.**
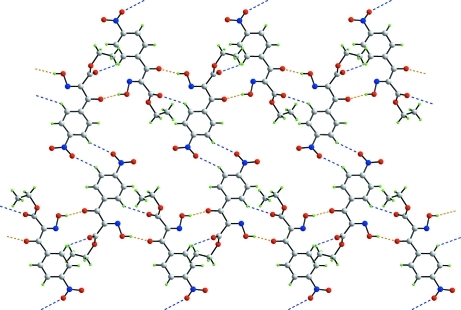
2-D array in (I) whereby the chains shown in Fig. 2 are reinforced by and are connected by C–H···O contacts (blue dashed lines). Colour code as for Fig. 2.

**Fig. 4.**
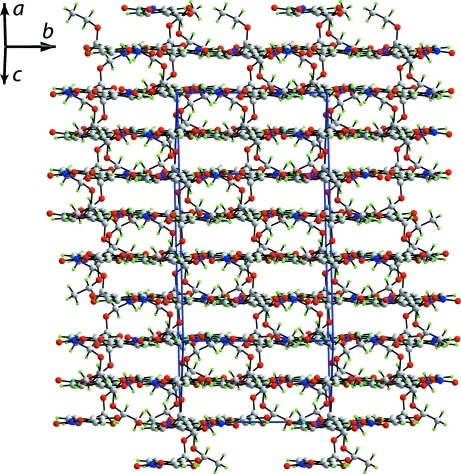
A view of the crystal packing in (I) showing the stacking of layers. Colour code as for Fig. 2.

## supplementary crystallographic information

### Comment

The title compound (I) was prepared as an intermediary during the synthesis of chiral hydroxyaminoacids and hydroxyaminoalcohols, as α-ketomethoxyimino compounds are reduced by sodium borohydride (Corrêa & Moran, 1999) and enantioselectively bio-reduced by whole cells of yeast (Kreutz *et al.*, 1997; Kreutz *et al.*, 2000).

The structure of (I) is non-planar as seen by the values of the C3–C4–C7–O3 and C4–C7–C8–N2 torsion angles within the central moiety of 8.8 (2) and 15.8 (2) °, respectively. The peripheral residues are twisted with respect to the inner atoms. Thus, the nitro ring is twisted out of the plane of the benzene ring to which it is connected: the C2–C1–N1–O1 torsion angle is -13.7 (3) °. Even more dramatic is the twist about the C8—C9 bond with the C7–C8–C9–O6 torsion angle being 94.77 (17)°, indicating the terminal ester group is orthogonal to the hydroxyimino residue. With respect to the C8═N2 bond, the conformation is *Z*. There are two other structures in the literature containing the basic C(═O)C(═NOH)C(═O)OC framework. In benzyl 2-(hydroxyimino)acetoacetate a *Z* conformation is found for the oxime group (Forsyth *et al.*, 2006) whereas in each of the two independent molecules comprising the asymmetric unit in ethyl 2-(hydroxyimino)-3-oxo-3-phenylpropionate, an *E* conformation is found (Ramos Silva *et al.*, 2004).

The crystal packing of (I) is dominated by O—H···O hydrogen bonding involving the oxime-O4—H and benzoyl-O3 atoms which leads to the formation of supramolecular chains along [0 1 0] with a flat topology, Fig. 2 and Table 1. The presence of C–H···O contacts provide stability to the chain. These C5–H···O5 contacts close 13-membered {···OC_3_O···HC_4_NOH} synthons, Fig. 3 and Table 1. The chains are linked into 2-D arrays in the [3 0 1] plane by further C–H···O contacts involving the nitro groups and centrosymmetric 10-membered {···ONC_2_H}_2_ synthons, Fig. 3 and Table 1. The resultant layer is essentially flat with the ethoxy groups lying above and below the layer. The methyl-H atoms of one of the disordered ethoxy groups form C–H···O contacts with the nitro-O1 atoms providing stability to the stacked layers, Fig. 4 and Table 1.

### Experimental

The title compound, (I), was prepared following a modified literature method (Adkins and Reeve, 1938). A solution of sodium nitrite (5 mmol) and water (2 ml) was added drop-wise to a solution of ethyl 3-oxo-3-(4-nitrophenyl)propanoate (2 mmol) in glacial acetic acid (3 ml) at 273 K. The resulting solution was stirred for 1 h at 273 K. The temperature was then raised to 303 K and the reaction left for a further hour. The reaction mixture was quenched with water (2.5 ml) and treated with ether (4 *x* 5 ml). The organic layer was dried with Mg(SO_4_) and the solvent evaporated to afford a mixture of *Z*:*E* (40:60) isomers in 87% yield that were separated by dissolving in ethyl acetate and precipitating with hexane.

### Refinement

The O– and C-bound H atoms were geometrically placed (O–H = 0.82 Å and C–H = 0.9–0.97 Å) and refined as riding with *U_iso_*(H) = 1.2*U_eq_*(C) and 1.*5U*_eq_(O and methyl-C).

Disorder was modelled for the ethyl group with two positions resolved for each of the C10 and C11 atoms. Fractional refinement (anisotropic) showed that the site occupancy factors were equal within experimental error and thus these were fixed at 0.5 in the final cycles of refinement.

### Crystal data

**Table:**

C_11_H_10_N_2_O_6_	*F*(000) = 1104
*M**_r_* = 266.21	*D*_x_ = 1.463 Mg m^−^^3^
Monoclinic, *C*2/*c*	Mo *K*α radiation, λ = 0.71073 Å
Hall symbol: -C 2yc	Cell parameters from 4194 reflections
*a* = 23.2347 (7) Å	θ = 27.5–1.0°
*b* = 12.0698 (6) Å	µ = 0.12 mm^−^^1^
*c* = 8.9698 (4) Å	*T* = 290 K
β = 106.100 (2)°	Irregular, colourless
*V* = 2416.82 (18) Å^3^	0.18 × 0.15 × 0.12 mm
*Z* = 8	

### Data collection

**Table:**

Nonius KappaCCD diffractometer	2004 reflections with *I* > 2σ(*I*)
Radiation source: fine-focus sealed tube	*R*_int_ = 0.033
graphite	θ_max_ = 27.5°, θ_min_ = 1.9°
CCD rotation images, thick slices scans	*h* = −30→28
8147 measured reflections	*k* = −15→14
2752 independent reflections	*l* = −8→11

### Refinement

**Table:**

Refinement on *F*^2^	Primary atom site location: structure-invariant direct methods
Least-squares matrix: full	Secondary atom site location: difference Fourier map
*R*[*F*^2^ > 2σ(*F*^2^)] = 0.048	Hydrogen site location: inferred from neighbouring sites
*wR*(*F*^2^) = 0.128	H-atom parameters constrained
*S* = 1.05	*w* = 1/[σ^2^(*F*_o_^2^) + (0.0493*P*)^2^ + 1.3639*P*] where *P* = (*F*_o_^2^ + 2*F*_c_^2^)/3
2752 reflections	(Δ/σ)_max_ < 0.001
189 parameters	Δρ_max_ = 0.20 e Å^−^^3^
0 restraints	Δρ_min_ = −0.19 e Å^−^^3^

### Special details

**Table:**

Geometry. All s.u.'s (except the s.u. in the dihedral angle between two l.s. planes) are estimated using the full covariance matrix. The cell s.u.'s are taken into account individually in the estimation of s.u.'s in distances, angles and torsion angles; correlations between s.u.'s in cell parameters are only used when they are defined by crystal symmetry. An approximate (isotropic) treatment of cell s.u.'s is used for estimating s.u.'s involving l.s. planes.
Refinement. Refinement of *F*^2^ against ALL reflections. The weighted *R*-factor *wR* and goodness of fit *S* are based on *F*^2^, conventional *R*-factors *R* are based on *F*, with *F* set to zero for negative *F*^2^. The threshold expression of *F*^2^ > 2σ(*F*^2^) is used only for calculating *R*-factors(gt) *etc*. and is not relevant to the choice of reflections for refinement. *R*-factors based on *F*^2^ are statistically about twice as large as those based on *F*, and *R*- factors based on ALL data will be even larger.

### Fractional atomic coordinates and isotropic or equivalent isotropic displacement parameters (Å^2^)

**Table:**

	*x*	*y*	*z*	*U*_iso_*/*U*_eq_	Occ. (<1)
O1	0.50114 (9)	0.16899 (16)	1.0271 (2)	0.1080 (7)	
O2	0.47793 (8)	0.32527 (15)	0.9163 (2)	0.0950 (6)	
O3	0.29936 (6)	−0.09600 (9)	0.44259 (15)	0.0585 (4)	
O4	0.21512 (6)	0.18581 (10)	0.13275 (15)	0.0581 (4)	
H4O	0.2168	0.2525	0.1169	0.087*	
O5	0.21223 (6)	−0.07843 (11)	0.10633 (16)	0.0652 (4)	
O6	0.15870 (6)	−0.01284 (12)	0.25941 (16)	0.0662 (4)	
N1	0.47188 (8)	0.22595 (16)	0.9220 (2)	0.0685 (5)	
N2	0.26049 (6)	0.15679 (11)	0.26243 (16)	0.0477 (3)	
C1	0.42724 (8)	0.17138 (15)	0.7937 (2)	0.0517 (4)	
C2	0.42939 (8)	0.05779 (16)	0.7813 (2)	0.0586 (5)	
H2	0.4580	0.0165	0.8527	0.070*	
C3	0.38815 (8)	0.00701 (15)	0.6605 (2)	0.0539 (4)	
H3	0.3893	−0.0695	0.6489	0.065*	
C4	0.34459 (7)	0.06915 (13)	0.55497 (18)	0.0426 (4)	
C5	0.34325 (8)	0.18320 (13)	0.5725 (2)	0.0489 (4)	
H5	0.3140	0.2249	0.5036	0.059*	
C6	0.38527 (8)	0.23516 (15)	0.6919 (2)	0.0537 (4)	
H6	0.3851	0.3118	0.7030	0.064*	
C7	0.30018 (7)	0.00492 (13)	0.43324 (19)	0.0434 (4)	
C8	0.25518 (7)	0.05606 (13)	0.29990 (18)	0.0423 (4)	
C9	0.20639 (7)	−0.02027 (13)	0.2087 (2)	0.0460 (4)	
C10	0.1039 (4)	−0.0815 (6)	0.1899 (9)	0.066 (2)	0.50
H10A	0.0680	−0.0394	0.1868	0.079*	0.50
H10B	0.1026	−0.1031	0.0849	0.079*	0.50
C11	0.1074 (2)	−0.1772 (4)	0.2855 (6)	0.0772 (13)	0.50
H11A	0.0750	−0.2264	0.2390	0.116*	0.50
H11B	0.1048	−0.1552	0.3863	0.116*	0.50
H11C	0.1448	−0.2143	0.2954	0.116*	0.50
C10A	0.1143 (2)	−0.0975 (4)	0.1926 (6)	0.093 (5)	0.50
H10C	0.1335	−0.1679	0.1858	0.112*	0.50
H10D	0.0907	−0.0761	0.0895	0.112*	0.50
C11A	0.0755 (3)	−0.1055 (8)	0.3013 (8)	0.127 (3)	0.50
H11D	0.0423	−0.1540	0.2578	0.190*	0.50
H11E	0.0607	−0.0333	0.3162	0.190*	0.50
H11F	0.0986	−0.1344	0.3993	0.190*	0.50

### Atomic displacement parameters (Å^2^)

**Table:**

	*U*^11^	*U*^22^	*U*^33^	*U*^12^	*U*^13^	*U*^23^
O1	0.1132 (14)	0.0968 (14)	0.0788 (11)	0.0063 (10)	−0.0320 (10)	−0.0089 (10)
O2	0.1013 (13)	0.0768 (12)	0.0881 (12)	−0.0268 (9)	−0.0050 (9)	−0.0171 (9)
O3	0.0739 (8)	0.0300 (6)	0.0639 (8)	−0.0020 (5)	0.0064 (6)	0.0004 (5)
O4	0.0661 (8)	0.0380 (6)	0.0606 (8)	0.0033 (6)	0.0019 (6)	0.0087 (6)
O5	0.0750 (9)	0.0528 (8)	0.0690 (9)	−0.0128 (6)	0.0219 (7)	−0.0214 (7)
O6	0.0567 (8)	0.0706 (9)	0.0725 (9)	−0.0230 (6)	0.0200 (7)	−0.0229 (7)
N1	0.0640 (10)	0.0740 (12)	0.0597 (10)	−0.0055 (9)	0.0045 (8)	−0.0137 (9)
N2	0.0525 (8)	0.0343 (7)	0.0530 (8)	−0.0005 (6)	0.0090 (6)	0.0020 (6)
C1	0.0474 (9)	0.0570 (11)	0.0489 (9)	−0.0046 (8)	0.0102 (7)	−0.0072 (8)
C2	0.0537 (10)	0.0581 (11)	0.0564 (11)	0.0083 (8)	0.0026 (8)	0.0019 (9)
C3	0.0572 (10)	0.0403 (9)	0.0599 (11)	0.0054 (8)	0.0090 (8)	0.0003 (8)
C4	0.0437 (8)	0.0358 (8)	0.0483 (9)	−0.0003 (6)	0.0127 (7)	−0.0003 (6)
C5	0.0513 (9)	0.0356 (8)	0.0553 (10)	0.0010 (7)	0.0071 (7)	−0.0010 (7)
C6	0.0604 (10)	0.0399 (9)	0.0581 (10)	−0.0047 (8)	0.0120 (8)	−0.0070 (8)
C7	0.0482 (9)	0.0328 (8)	0.0496 (9)	−0.0010 (7)	0.0144 (7)	−0.0007 (6)
C8	0.0463 (8)	0.0328 (8)	0.0474 (9)	−0.0017 (6)	0.0126 (7)	−0.0028 (6)
C9	0.0515 (9)	0.0344 (8)	0.0492 (9)	−0.0023 (7)	0.0089 (7)	0.0022 (7)
C10	0.043 (2)	0.057 (3)	0.087 (5)	−0.016 (2)	0.001 (3)	−0.004 (3)
C11	0.068 (3)	0.074 (3)	0.081 (3)	−0.027 (2)	0.005 (2)	0.007 (3)
C10A	0.061 (5)	0.139 (10)	0.085 (6)	−0.044 (5)	0.031 (4)	−0.056 (6)
C11A	0.105 (5)	0.187 (8)	0.105 (4)	−0.088 (5)	0.057 (4)	−0.057 (5)

### Geometric parameters (Å, °)

**Table:**

O1—N1	1.212 (2)	C4—C7	1.494 (2)
O2—N1	1.210 (2)	C5—C6	1.384 (2)
O3—C7	1.2215 (19)	C5—H5	0.9300
O4—N2	1.3809 (18)	C6—H6	0.9300
O4—H4O	0.8200	C7—C8	1.487 (2)
O5—C9	1.193 (2)	C8—C9	1.513 (2)
O6—C9	1.312 (2)	C10—C11	1.427 (10)
O6—C10A	1.458 (5)	C10—H10A	0.9700
O6—C10	1.502 (6)	C10—H10B	0.9700
N1—C1	1.474 (2)	C11—H11A	0.9600
N2—C8	1.276 (2)	C11—H11B	0.9600
C1—C6	1.372 (3)	C11—H11C	0.9600
C1—C2	1.378 (3)	C10A—C11A	1.503 (8)
C2—C3	1.376 (3)	C10A—H10C	0.9700
C2—H2	0.9300	C10A—H10D	0.9700
C3—C4	1.397 (2)	C11A—H11D	0.9600
C3—H3	0.9300	C11A—H11E	0.9600
C4—C5	1.387 (2)	C11A—H11F	0.9600
			
N2—O4—H4O	109.5	N2—C8—C9	123.39 (14)
C9—O6—C10A	112.3 (3)	C7—C8—C9	115.87 (13)
C9—O6—C10	121.3 (4)	O5—C9—O6	126.44 (16)
O2—N1—O1	123.25 (18)	O5—C9—C8	123.10 (16)
O2—N1—C1	118.35 (18)	O6—C9—C8	110.45 (14)
O1—N1—C1	118.39 (19)	C11—C10—O6	107.2 (6)
C8—N2—O4	110.88 (13)	C11—C10—H10A	110.3
C6—C1—C2	122.63 (16)	O6—C10—H10A	110.3
C6—C1—N1	119.02 (17)	C11—C10—H10B	110.3
C2—C1—N1	118.35 (17)	O6—C10—H10B	110.3
C3—C2—C1	118.33 (17)	H10A—C10—H10B	108.5
C3—C2—H2	120.8	C10—C11—H11A	109.5
C1—C2—H2	120.8	C10—C11—H11B	109.5
C2—C3—C4	120.66 (17)	H11A—C11—H11B	109.5
C2—C3—H3	119.7	C10—C11—H11C	109.5
C4—C3—H3	119.7	H11A—C11—H11C	109.5
C5—C4—C3	119.42 (16)	H11B—C11—H11C	109.5
C5—C4—C7	124.40 (15)	O6—C10A—C11A	105.2 (4)
C3—C4—C7	116.12 (15)	O6—C10A—H10C	110.7
C6—C5—C4	120.29 (16)	C11A—C10A—H10C	110.7
C6—C5—H5	119.9	O6—C10A—H10D	110.7
C4—C5—H5	119.9	C11A—C10A—H10D	110.7
C1—C6—C5	118.66 (16)	H10C—C10A—H10D	108.8
C1—C6—H6	120.7	C10A—C11A—H11D	109.5
C5—C6—H6	120.7	C10A—C11A—H11E	109.5
O3—C7—C8	116.64 (14)	H11D—C11A—H11E	109.5
O3—C7—C4	119.20 (15)	C10A—C11A—H11F	109.5
C8—C7—C4	124.15 (14)	H11D—C11A—H11F	109.5
N2—C8—C7	120.63 (14)	H11E—C11A—H11F	109.5
			
O2—N1—C1—C6	−14.5 (3)	O4—N2—C8—C7	177.96 (13)
O1—N1—C1—C6	166.2 (2)	O4—N2—C8—C9	1.9 (2)
O2—N1—C1—C2	165.6 (2)	O3—C7—C8—N2	−165.30 (16)
O1—N1—C1—C2	−13.7 (3)	C4—C7—C8—N2	15.8 (2)
C6—C1—C2—C3	0.9 (3)	O3—C7—C8—C9	11.0 (2)
N1—C1—C2—C3	−179.25 (16)	C4—C7—C8—C9	−167.90 (14)
C1—C2—C3—C4	−1.1 (3)	C10A—O6—C9—O5	8.9 (3)
C2—C3—C4—C5	0.2 (3)	C10—O6—C9—O5	0.4 (4)
C2—C3—C4—C7	−176.95 (16)	C10A—O6—C9—C8	−170.7 (2)
C3—C4—C5—C6	1.0 (3)	C10—O6—C9—C8	−179.1 (3)
C7—C4—C5—C6	177.94 (15)	N2—C8—C9—O5	91.4 (2)
C2—C1—C6—C5	0.3 (3)	C7—C8—C9—O5	−84.8 (2)
N1—C1—C6—C5	−179.55 (16)	N2—C8—C9—O6	−89.0 (2)
C4—C5—C6—C1	−1.3 (3)	C7—C8—C9—O6	94.77 (17)
C5—C4—C7—O3	−168.26 (17)	C9—O6—C10—C11	96.0 (7)
C3—C4—C7—O3	8.8 (2)	C10A—O6—C10—C11	53.9 (18)
C5—C4—C7—C8	10.7 (2)	C9—O6—C10A—C11A	159.5 (4)
C3—C4—C7—C8	−172.33 (15)	C10—O6—C10A—C11A	−59 (2)

### Hydrogen-bond geometry (Å, °)

**Table:**

*D*—H···*A*	*D*—H	H···*A*	*D*···*A*	*D*—H···*A*
O4—H4o···O3^i^	0.82	1.91	2.7165 (16)	166
C5—H5···O5^i^	0.93	2.58	3.371 (2)	144
C2—H2···O1^ii^	0.93	2.55	3.393 (3)	151
C11—H11a···O1^iii^	0.96	2.52	3.426 (5)	158

Symmetry codes: (i) −*x*+1/2, *y*+1/2, −*z*+1/2; (ii) −*x*+1, −*y*, −*z*+2; (iii) *x*−1/2, *y*−1/2, *z*−1.
